# Procedural fairness in ethnic-cultural decision-making: fostering social cohesion by incorporating minority and majority perspectives

**DOI:** 10.3389/fpsyg.2023.1025153

**Published:** 2023-07-04

**Authors:** Kim Dierckx, Alain Van Hiel, Barbara Valcke, Kees van den Bos

**Affiliations:** ^1^Department of Developmental, Personality and Social Psychology, Faculty of Psychology and Educational Sciences, Ghent University, Ghent, East Flanders, Belgium; ^2^School of Law, Faculty of Law, Economics, and Governance, Utrecht University, Utrecht, Netherlands

**Keywords:** procedural fairness, ethnic-cultural minority groups, collective model, minority collective identity, moral obligations

## Abstract

Recent research describes how procedural fairness can be used to resolve issues related to ethnic-cultural matters. The central finding in this strand of literature is that when minority members experience procedurally fair treatment by societal actors regarding ethnic-cultural issues, this will lead to a range of outcomes that are beneficial for social cohesion. Although these results are promising, it remains yet to be shown that such group-specific treatment fairness does not hamper social cohesion by inciting misapprehension among members of non-recipient groups. Therefore, the present study set out to examine two central questions. First, how would *minority* group members respond to treatment fairness of citizens belonging to another minority group? Second, how would *majority* group members respond to treatment fairness of citizens belonging to minority groups? Two experimental studies (total *N* = 908) examined these questions. In Study 1, we compared ethnic-cultural minorities’ reactions to procedurally (un)fair treatment of their own versus a different minority group. In Study 2, we compared minority and majority group members’ responses to procedurally (un)fair treatment of minority group members. Results show that minority group member reactions to ethnic-cultural procedural fairness emanate from a shared bond with the fairness recipient(s) of the other minority group. Conversely, majority group members’ reactions are driven primarily by a perceived moral obligation to act rightfully toward members of disadvantaged groups. Taken together, our results suggest that ethnic-cultural procedural fairness enactment fosters societal unity among different groups, possibly strengthening social cohesion for well-being and prosperity among members of these groups.

## 1. Introduction

Social cohesion, defined as “the degree of connectedness among groups in society” (Manca, 2014, p. 20), is commonly regarded as a quintessential building block of a stable and harmonious society. A plethora of studies have empirically corroborated this laymen’s premise, linking social cohesion to lower crime rates (e.g., Alesina and La Ferrara, 2000; Letki, 2008), more effective democracy and governance (e.g., Putnam, 1993), better individual and public health (e.g., Kawachi and Berkman, 2000; Chuang et al., 2013) and enhanced wellbeing (e.g., Elands et al., 2018; Florez et al., 2020). Bearing these observations in mind, it can be deemed alarming that recent research is showing a steady decline in social cohesion (Putnam, 2000, 2007; Council of Europe, 2005; Schmeets and te Riele, 2014; Borkowska and Laurence, 2021). In order to provide societal actors (e.g., local authorities, the courts, political stakeholders, the police) with the relevant knowledge and the necessary practical tools to counter this trend, more empirical focus on interventions and policies which build, preserve, and strengthen cohesion within societies thus seems pivotal. Therefore, in the present research, we aim to provide a critical examination of one such intervention which has been related to various aspects of social cohesion: Applying procedural fairness in ethnic-cultural decision-making.

Procedural fairness – or “treatment fairness” hereafter – refers to the perceptions of fair procedures implemented to arrive at a particular outcome (Thibaut and Walker, 1975; Tyler and Lind, 1992; Lind, 2001; Van den Bos et al., 2001). These perceptions are most likely to arise when authorities make their decisions in an ethical way, without a priori favoring one party over the other, relying on accurate information, and granting those affected by the outcome the opportunity to voice their opinion (Thibaut and Walker, 1975; Leventhal, 1980; Tyler and Lind, 1992). Of special relevance for the present research, a new line of research has emerged, documenting how procedural fairness can be applied specifically to ethnic-cultural decision-making – which has been defined as “resolving issues related to ethnic-cultural, religious or linguistic matters” (Valcke et al., 2020a). These issues include – but are not limited to – whether to allow minority employees to wear a headscarf in the workplace, the possible removal of statues reminiscent of colonial times, and addressing language discrimination at schools. The central tenet of this recent strand of literature is that the perception among minority group members of treatment fairness regarding ethnic-cultural issues by societal actors is associated with a range of outcomes that are beneficial to social cohesion. Specifically, this research has consistently revealed that procedural fairness perceptions vis-à-vis ethnic-cultural issues enhance social trust (Valcke et al., 2020b) and trust in the national majority group (Dierckx et al., 2021), fosters sense of societal belonging (Valcke et al., 2020b) and societal identification (Valcke et al., 2020a), and strengthen perceptions of democratic legitimacy of societal actors (Dierckx et al., 2020) among members of the fairness recipient minority group. Conversely, a lack of such perceptions has been associated with greater sense of discrimination toward one’s minority group (Dierckx et al., 2021) and lower feelings of social acceptance (Valcke et al., 2020b). Taken together, these findings illustrate that the application of procedural fairness in the resolution of ethnic-cultural issues opens new horizons for societal actors to bolster social cohesion.

Yet, an important caveat should be mentioned. The existing body of research described above can also be problematized because it has left two critical questions unanswered. Firstly, how would minority group members respond to fair or unfair treatment of citizens belonging to another minority group? Secondly, how would majority group members respond to treatment fairness of citizens belonging to minority groups? These are relevant questions to ask, because it may very well be that the benign effects of ethnic-cultural procedural fairness are limited to members of the group which is recipient of the fair treatment. Furthermore, overt displays of procedural fairness enacted toward a given group may elicit negative reactions among members of non-recipient groups because these groups can believe that such fairness efforts are irreconcilable with their own interests. At any rate, the gap in the literature on bystander responses to fairness is problematic because, in the absence of information about the perspectives of members of uninvolved societal groups, the “net” effect of applying procedural fairness to decisions vis-à-vis ethnic-cultural issues cannot accurately be assessed. That is, if research were to show that majority group members may, on average, oppose procedural fairness in ethnic-cultural decision-making, the “net” effect of fair procedures vis-à-vis ethnic-cultural issues would in fact be negative (i.e., a decrease in social cohesion would be observed). As such, to further empirically validate the assumption that ethnic-cultural procedural fairness is a useful “tool to bridge intergroup disputes and to ameliorate intergroup relations” (Dierckx et al., 2021, p. 345) or the notion that it is a “medium to prevent the bankruptcy of social capital” (Dierckx et al., 2021, p. 356), it is thus critical to take the perspective and viewpoint of all societal stakeholders into account – both fairness recipients and non-recipients. Hence, in the present research, we address the above questions, and we aim to disentangle the psychological mechanisms that give rise to the observed procedural fairness effects among uninvolved minority and majority groups. Specifically, we show that minority group members’ reactions to ethnic-cultural procedural fairness are driven by a shared bond with the recipients – based on their minority group membership – which is engrained in their collective self (i.e., those aspects of the self that are based on memberships in large social groups; Sedikides and Brewer, 2015; Nehrlich et al., 2019). Furthermore, we also pit two alternative explanatory accounts for majority group members’ reactions to ethnic-cultural procedural fairness against one another. The first account, grounded in deontic theory (Folger, 2001; Beugré, 2012; Barclay et al., 2017), predicts that, on average, majority group members’ responses are driven by the universal moral obligation to act rightfully toward other people in general. By contrast, the second account, based on intergroup relations research (Dierckx et al., 2022), proposes that advantaged group members’ behavior toward disadvantaged groups is shaped by the belief that a privileged societal status entails the moral responsibility to treat disadvantaged groups with utmost care and diligence. In sum, by probing the reactions of non-involved social groups to ethnic-cultural procedural fairness and disentangling the underlying psychological processes, the present research further illustrates the potential of procedural fairness as a promising tool to foster social cohesion.

### 1.1. Minority members’ responses to fair treatment of members of another minority group

As we outlined in the previous section, a first question that needs to be addressed and which we will try to answer in the present research is: How would minority group members respond to treatment fairness of citizens *belonging to another minority group*? At first sight, one may contend that ethnic-cultural issues involve only a single minority group at once. Therefore, they do not affect the interest of members of minority groups other than the one implicated by the fair treatment; and hence, no effects of procedural fairness enactment should be expected. However, in the present research, we explore an alternative possibility and argue that ethnic-cultural procedural fairness perceptions will in fact elicit similar responses among members of involved and non-involved minority groups.

To ground our predictions we build on Valcke et al.’s (2020b) collective model of procedural fairness. This model focuses on the psychological impact of ethnic-cultural procedural fairness on members of fairness recipients groups. Specifically, the framework states that ethnic-cultural procedural fairness activates representations embedded in the collective self (which allude to being a member of the fairness recipient minority group). As a consequence, the fair treatment of one’s minority group – even when this treatment does not involve the person him or herself but a fellow minority member – reflects positively on the individual minority group member (leading to the observed benign effects for social cohesion).

**Figure 1:**
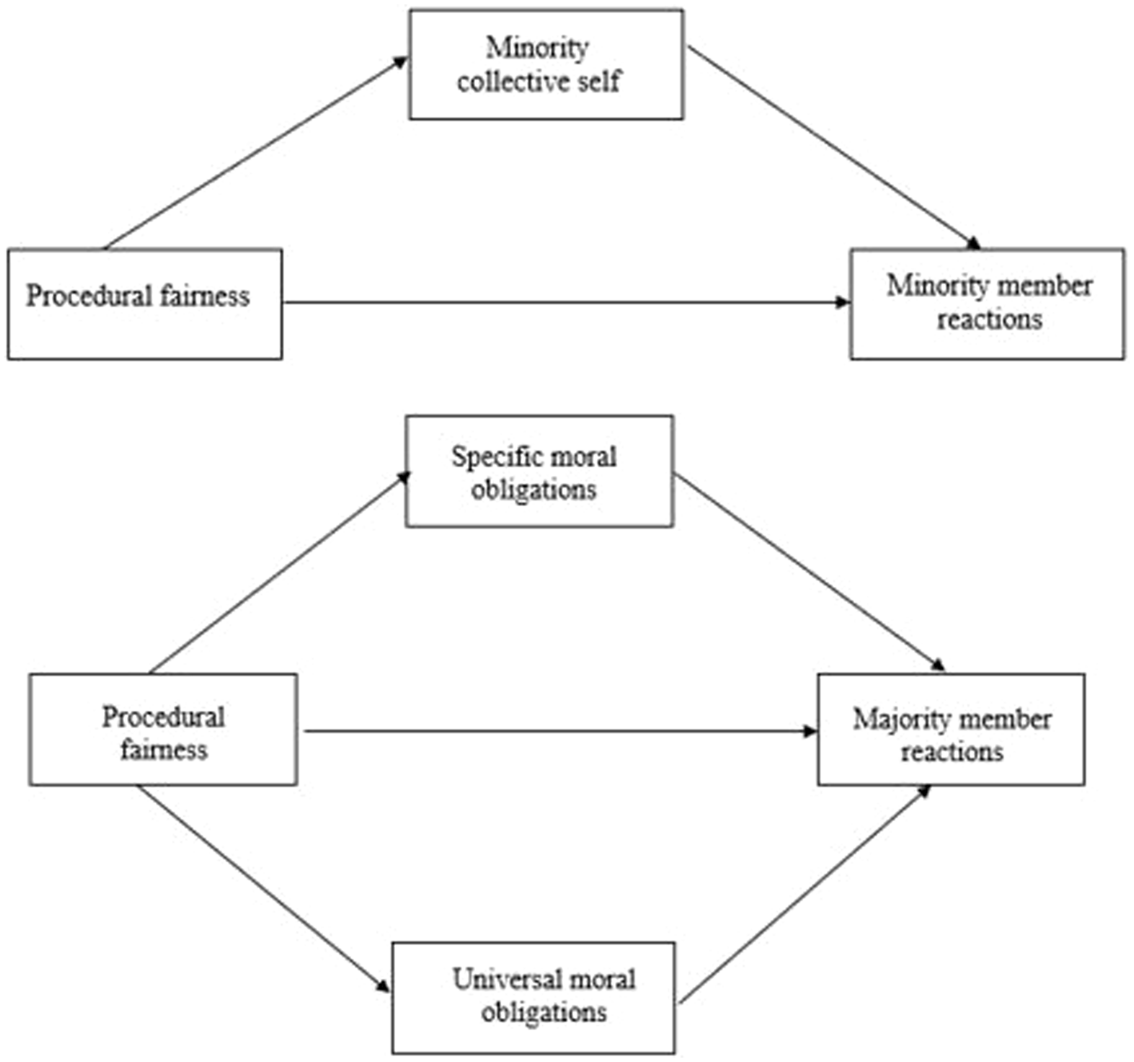
Hypothesized models of psychological processes giving rise to minority group member (top panel) and majority group member (bottom panel) reactions to ethnic-cultural procedural fairness enactment vis-à-vis minority groups by societal actors.

In this vein, it could be argued that witnesses of procedurally fair and unfair treatment of citizens belonging to a *different* minority group also share a critical attribute with the recipient of the (un)fair treatment. That is, although they are not members of the *same* minority group, witnesses and recipients here have in common that they all are *a member of a minoritized group as such*. And, it is this shared “disadvantaged group membership” – which should be embedded in their collective self as well – that could create the ingroup context which then gives rise to analogous ethnic-cultural procedural fairness effects. In other words, the perception of treatment fairness of people belonging to a different minority group could “activate” aspects of the collective self, alluding to being a member of a minority group as such (“minority collective identity” hereafter). Consequently, and analogous to members of the recipient group, one could anticipate that treatment fairness of people belonging to a different minority group should sort effects similar to those observed when the own minority group is involved (*Hypothesis 1*). Furthermore, we also expected that minority collective identity would mediate these effects among members of non-recipient minority groups (*Hypothesis 2*). The top panel of Figure 1 depicts an overview of our hypothesized model among minority members.

### 1.2. Majority members’ responses to ethnic-cultural procedural fairness

The present contribution also aimed to address a second question which arises from the collective model. That is, just like members of non-involved minority groups, majority group members are neither targeted nor directly affected by ethnic-cultural decision-making (and its outcomes). However, unlike their minority counterparts, majority group members do not share any ties with the fairness recipients based on shared membership of a societal disadvantaged group. Thus, majority group members can be nothing but mere passive witnesses to the type of procedurally fair treatment we have described so far. Of course, this does not exclude them from developing their own opinions about and reactions toward ethnic-cultural procedural fairness. Hence, a second relevant question is: How would *majority group members* respond to procedurally fair and unfair treatment of citizens belonging to minority groups?

Recent literature provides some relevant insights here. During the last decade, justice scholars have shifted their focus away from the consequences toward the antecedents of procedural fairness perceptions – a trend which has been coined “the fifth wave” in fairness research (Brockner and Wiesenfeld, 2019). This approach has yielded the insight that fairness perceptions are a form of “motivated cognition” (Barclay et al., 2017), a subjective experience shaped by individual and environmental factors (Folger and Glerum, 2015). Within this approach, special attention has been devoted to the role of moral motives. For example, deontic accounts have been put forth (see Folger, 2001; Beugré, 2012; Barclay et al., 2017). These accounts contend that any behavior that conforms to norms of universal moral obligations, both for oneself and for others, is perceived as “fair,” and hence, is appraised in a positive way. Thus, according to deontic theory, people should care about fairness enactment toward social groups to which they are not affiliated because it aligns with the universal moral obligation to act rightfully toward all humanity (Folger, 2001; Barclay et al., 2017). In line with this reasoning, Colquitt (2004) revealed that people’s fairness perceptions are not only shaped by their own experiences, but also by those of peers, and that the most positive effects occur when both types of fairness perceptions are high. In a similar vein, Dierckx et al. (2023a) studied employees’ responses to procedurally fair treatment of the minority workforce by organizational decision-makers. Interestingly, these scholars did not observe any notable differences in the reactions of minority and majority group employees (in terms of organizational identification and job satisfaction). Hence, based on these premises and empirical findings, we anticipated to observe similar effects of ethnic-cultural procedural fairness among majority group members (compared to their minority counterparts; *Hypothesis 3*).

Additionally, the present research also aimed to move beyond the observations made by Dierckx et al. (2023a). Although these scholars’ results are interesting in their own right, it must be noted that they do not allow inferences about the psychological motivators underlying the observed behavior among majority group members. As noted above, it may well be that their responses are grounded in deontic motives (i.e., *universal moral obligations*) and thus align with the predictions of the deontic account. However, in the present research, we explored yet another reason for majority members’ hypothesized positive responses to ethnic-cultural procedural fairness.

To explain our theoretical rationale, we refer to a recent study by Dierckx et al. (2022). These authors found that treatment of disadvantaged groups is particularly weighed and judged in terms of adherence to moral obligations. Specifically, their results revealed that majority group third-party witnesses implicitly take into account the power differentials between agents and their targets, and consequently, advantaged agents are subjected to strict and elevated moral expectations with respect to their conduct toward disadvantaged groups (compared to a disadvantaged agent and advantaged target groups; Dierckx et al., 2022). Based on this premise, we hypothesize that majority members’ reactions to ethnic-cultural procedural fairness may also be driven by *specific moral obligations* (to act rightfully and unbiasedly toward disadvantaged groups), and not only by the sheer universal moral obligations (to act rightfully and unbiasedly toward “all humanity”) encapsulated in the deontic framework. As such, we formulated a specific moral obligations account which mirrors the deontic account in predicting positive responses to the perception of ethnic-cultural procedural fairness among the national majority. However, unlike its deontic counterpart, our specific moral obligations account assumes these reactions are primarily driven by moral obligations to act rightfully and unbiasedly toward disadvantaged groups specifically. In sum, given that we did not have any a priori reason to favor one explanatory account over the other, we expected that both universal and specific moral obligations would mediate the effects of ethnic-cultural procedural fairness perceptions among majority group members (*Hypothesis 4*). The bottom panel of Figure 1 shows an overview of our hypothesized model of majority member reactions.

## 2. The present studies

In sum, the central goals of the present research were (1) to examine the responses of members of non-involved minority groups and majority group members to instances of ethnic-cultural procedural fairness, and (2) to unravel the underlying psychological processes. To this end, two studies were conducted. In Study 1, we sampled Hispanic American minority group members and gauged their reactions to procedurally (un)fair treatment of a fellow minority group member (a Hispanic American citizen) or a member of another minority group (a Black American citizen). Moreover, and in line with our theoretical reflections based on the collective model by Valcke et al. (2020b), we additionally explored the mediating role of minority collective identity in our participants’ responses. Then, in Study 2, we sampled both Black American minority group members and White American majority group members, and we gauged their reactions to procedurally (un)fair treatment of a Black American citizen. Furthermore, we additionally investigated the mediating role of the three psychological processes we outlined in the Introduction (collective identity for Black participants, and universal and specific moral obligations for White participants).

Importantly, for the present purposes, we focused on positive emotional reactions to ethnic-cultural procedural fairness as our main dependent variable. Our reasons for doing so were twofold. First, emotions have been shown to be key drivers of procedural fairness effects (e.g., van Zomeren et al., 2004; Murphy and Tyler, 2008). Secondly and more importantly, a positive emotional reaction to ethnic-cultural procedural fairness among bystanders can also (indirectly) be linked to social cohesion. For example, institutional trust has been coined one of the key dimensions of social cohesion by various authors (e.g., Dragolov et al., 2016). It stands to reason that a positive emotional state as a consequence of procedural justice perceptions may also foster trust in the decision-making institutions among bystanders – thereby bolstering social cohesion. Furthermore, another core dimension of social cohesion is the degree of trust and reciprocity among members of a given society (Delhey and Dragolov, 2016; Delhey et al., 2018). And, obviously, positive emotional reactions to procedural justice processes that do not involve the individual him or herself can be deemed reflective of high quality social relations between a society’s members. Thus, from this perspective too, positive emotional reactions can be coined a relevant “proxy variable” which can inform us about the effects of bystander procedural fairness perceptions on social cohesion.

The materials, data files, and data scripts of both studies can be accessed through our Open Science webpage.^1^ The research was conducted according to the ethical rules presented in the General Ethical Protocol of our faculty. All measures, manipulations and exclusions are reported.

## 3. Study 1

### 3.1. Method

#### 3.1.1. Participants

Study 1 was preregistered at https://aspredicted.org/6KL_S3D. Sample size was determined prior to data collection. Given that the main objective of Study 1 was to study the reactions of uninvolved minority group members to ethnic-cultural procedural fairness, and more specifically the mediating role of minority collective self therein, we based our power calculations on the indirect effect of this variable obtained in a pilot study [i.e., *b* = 0.10 (0.012, 0.178); see Supplementary online materials on our open science webpage]. Hence, we simulated 10,000 times a model wherein minority collective self mediated the relationship between procedural fairness and emotional reactions (i.e., our outcome variable, see below). The regression coefficients of our pilot study were used as estimates for the direct paths in the simulations. The results of these calculations revealed that, assuming standard criteria (*α* = 0.05), a sample of *N* = 400 participants would yield over 99% power to detect an indirect effect of the aforementioned magnitude. Anticipating some dropout, we slightly oversampled and recruited 430 Hispanic American minority group members^2^ on Prolific. Thirty-one participants were excluded because they failed the complainant’s ethnic background attention check (“What was the ethnic group of the complainant?”). Hence, our final sample consisted of 399 participants (145 males, *M* = 30.27, *SD* = 10.58, range = 18–73).

#### 3.1.2. Procedure

Prolific workers were invited to participate in a study about “social opinions.” They then read a fictitious news article describing the court case of an American citizen who was suing his employer for not allowing him to wear his Christian cross necklace in the workplace. It was conveyed that, although employers cannot typically ban articles of religious significance from being worn at work, the packaging factory where the man worked argued that the necklace could have represented “a safety hazard” at the production line, and thus, they forbade him from wearing it. Critically, in the current study, we employed a full 2×2 design, wherein we manipulated both procedural fairness (fair vs. unfair) and the ethnic group of the complainant (Hispanic or own minority group vs. Black or other minority group). Thus, participants were randomly assigned to one out of four conditions: fair/own minority group (*n* = 97), fair/other minority group (*n* = 104), unfair/ own minority group (*n* = 95), unfair/other minority group (*n* = 103). In the fair procedure conditions, participants read that the judge had allowed both the complainant and the defendant party to voice their views and to present the reasons behind them. Conversely, in the unfair procedure conditions, the judge had refrained from making such efforts. Moreover, because procedural fairness yields the strongest effects when distributive fairness is low (Brockner and Wiesenfeld, 1996, 2005), we added a sentence explaining that the defendant party would not be prosecuted. Subsequently, participants completed three manipulation checks, our attention check, and dependent measures. Finally, they were debriefed and thanked for their cooperation.

#### 3.1.3. Measures

To gauge participants’ *positive emotional reactions* to the news article, we implemented six items, which we adapted from Stouten et al. (2005). Having read the news article, participants were asked to indicate the extent to which they felt “angry,” “irritated,” “disappointed,” “happy,” “elated” and “relieved” (scaled 1 = “not at all” to 5 = “very much”). The former three items were recoded such that higher scores on all items reflected more positive emotional reactions. Then, the entire itemset was aggregated into a single, reliable measure (*M* = 2.64, *SD* = 0.85, *α* = 0.82). Participants were also asked to indicate for a series of statements the extent to which they applied as a reason for their (lack of) positive emotional reactions (scaled 1 = “not applicable at all” to 5 = “strongly applicable”). Amongst some filler items, we included six items referring to participants’ *minority collective self* as a reason to have positive emotional reactions, e.g., “The news article made me feel this way because the treatment of the complainant… reflects on me being a member of a minority group,” and “… leaves me indifferent because I do not think of myself in terms of minority group membership” (reverse-scored). These items were based on Leach et al.’ (2008) in-group identification measure, and more specifically, on the items measuring the “self-definition” component. The collective self is defined as those aspects of the self that relate to “membership in large social groups” (Sedikides and Brewer, 2015), and being a minority member as such should be one salient aspect for Hispanic Americans (having read the article). Furthermore, perceiving oneself as a member of a given group inevitably entails “a self-categorization that includes the individual in that group” (Tajfel, 1978). Hence, we operationalized minority collective self – as a reason for positive emotional reactions – as the extent to which participants self-categorized as a minority person (see also Hogg and Williams, 2000). The reliability of this scale was adequate (*M* = 2.97, *SD* = 0.91, *α* = 0.80).

### 3.2. Data analysis and results

#### 3.2.1. Manipulation checks

A two-way ANOVA with procedural fairness (fair vs. unfair) and fairness recipient (own vs. other minority group) on the first manipulation check (“According to the news article, the decision was made in a fair and unbiased way”) revealed a main effect of procedural fairness [*F* (1, 394) = 512.28, *p* < 0.001, *η^2^* = 0.56]: Participants in the fair procedure conditions (*M* = 4.56, *SD* = 0.90) displayed stronger agreement with this statement compared to those in the unfair procedure conditions (*M =* 1.89, *SD* = 1.40, *d* = 2.27). Neither the main effect of fairness recipient, nor the procedural fairness*fairness recipient interaction reached significance (both *F*s < 1.43, *p*s > 0.308). Analogously, for the second item (“According to the news article, the judge allowed the complainant to voice his opinions”) participants were also more likely to agree in the fair procedure conditions (*M =* 4.50, *SD* = 1.02) vs. the unfair procedure conditions [*M =* 1.41, *SD* = 0.91, *d =* 3.20; *F* (1, 394) = 1011.46, *p < 0*.001, *η^2^ = *0.71]. As before, neither the main effect of fairness recipient, nor the procedural fairness*fairness recipient interaction reached significance (both *F*s < 0.84, *p*s > 0.362). Conversely, for the third item (“The news article referred to the decision by a US court not to allow a Latino employee to wear a cross in the workplace”), we obtained a main effect of fairness recipient (*F* (1, 394) = 1189.83, *p* < 0.001, *η^2^* = 0.75): Participants in the own minority group conditions (*M* = 4.67, *SD* = 0.80) displayed stronger agreement with this statement compared to those in the other minority group conditions (*M =* 1.44, *SD* = 1.05, *d* = 3.47). Neither the main effect of procedural fairness, nor the procedural fairness*fairness recipient interaction reached significance (both *F*s < 0.69, *p*s > 0.410). As such, we concluded that our manipulations had both been successful.

#### 3.2.2. Dependent variables

We first explored participants’ positive emotional reactions after reading the news article and their references to collective self as a reason to have positive emotional reactions. Overall, participants reported rather neutral emotional reactions [*M* = 2.64, *SD* = 0.85, *t* (396) = 3.35, *p* = 0.999]. By contrast, their ratings of minority collective self as a reason for their emotional reactions were significantly above-average [*M* = 2.97, *SD* = 0.91, *t* (395) = 10.14, *p* < 0.001]. Both variables also correlated significantly and negatively (*r* = −0.50, *p* < 0.001).

To further investigate the effects of our manipulation, we ran a two-way MANOVA with procedural fairness (fair vs. unfair) and fairness recipient (own vs. other minority group) as between-subject factors, and minority collective self and positive emotional reactions as the dependent variables. The omnibus MANOVA test yielded a significant main effect of procedural fairness [*F* (2, 391) = 32.78, *p* < 0.001, *η^2^* = 0.14]. Follow-up Welch’s *t*-tests revealed significant differences between the fair procedure and the unfair procedure conditions for minority collective self [*M*_fair_ = 2.80, *SD*_fair_ = 0.83; *M*_unfair_ = 3.14, *SD*_unfair_ = 0.97; *F* (1, 383) = 14.0, *p* < 0.001] and positive emotional reactions [*M*_fair_ = 2.96, *SD*_fair_ = 0.75; *M*_unfair_ = 2.32, *SD*_unfair_ = 0.83; *F* (1, 389) = 65.2, *p* < 0.001]. Neither the main effect of fairness recipient, nor the procedural fairness*fairness recipient interaction reached significance (both *F*s < 1.40, *p*s > 0.251).

#### 3.2.3. Bayesian analysis

To further substantiate our claim that treatment fairness of people belonging to a different minority group should sort effects similar to those observed when the own minority group is involved (i.e., *Hypothesis 1*), we additionally calculated Bayes factors (*BF*s) for the critical procedural fairness*fairness recipient interactions (dependent variables = minority collective self, positive emotional reactions). One specific advantage that Bayesian analyses hold over their frequentist counterparts is that they can provide evidence in favor of two competing hypotheses, e.g., the null hypothesis (*H_0_*) and the alternative hypothesis (*H_a_*). As such, because *Hypothesis 1* essentially argues in favor of a procedural fairness*fairness recipient null effect, we reasoned that Bayes factor estimation could thus provide important additional information about the validity of this null effect claim, on top of what our frequentist analyses in the previous section could reveal.

Typically, *BF*s quantify the likelihood of the obtained data given *H_a_* relative to the likelihood of the data given *H_0_*. Consequently, *BF*s for the *H_a_* < 1 mean that the data is more likely under the *H_0_* while *BF*s > 1 indicate that the data is more likely under the alternative hypothesis. As a rule of thumb, Wetzels and Wagenmakers (2012, based on Jeffreys, 1961) proposed that *BF*s ranging from 1–3 can be interpreted as providing “anecdotal evidence” in favor of the tested hypothesis (either *H_0_* or *H_a_*), whereas Bayes factors higher than three indicate “substantial evidence” for the tested hypothesis.

The results of our Bayesian analyses revealed that the *BF*s for the procedural fairness*fairness recipient interaction effects on participants’ (minority) collective self and positive emotional reactions (i.e., the alternative hypothesis) were 0.17 and 0.19, respectively. Or, in other words, the *BF*s favoring the null hypothesis were 5.88 and 5.26, suggesting substantial evidence for the null hypothesis. Thus, taken together with our frequentist results, our Bayesian analyses further validated our expectation that treatment fairness of people belonging to a different minority group likely sorts effects similar to those observed when the own minority group is involved.

#### 3.2.4. Mediation analysis

To investigate the role of minority collective self as an explanatory process variable, we conducted a mediation analysis, using the *Lavaan* package (Rosseel, 2012) in R Core Team (2021), with procedural fairness (dummy-coded: fair = 1, unfair = 0) and fairness recipient (own minority group = 1, other minority group = 0) as independent variables, minority collective self as mediator and positive emotional reactions as outcome. In line with our hypothesis, the results of this analysis indeed revealed that minority collective self mediated the relationship between procedural fairness and positive emotional reactions [*b* = 0.14 (0.063, 0.218), *SE* = 0.040, *p* < 0.001]. The indirect effect of fairness recipient (through minority collective self) on positive emotional reactions did not reach significance [*b* = −0.01 (−0.083, 0.064), *SE* = 0.037, *p* = 0.806].^3^

#### 3.2.5. Moderated mediation analysis

**Figure 2:**
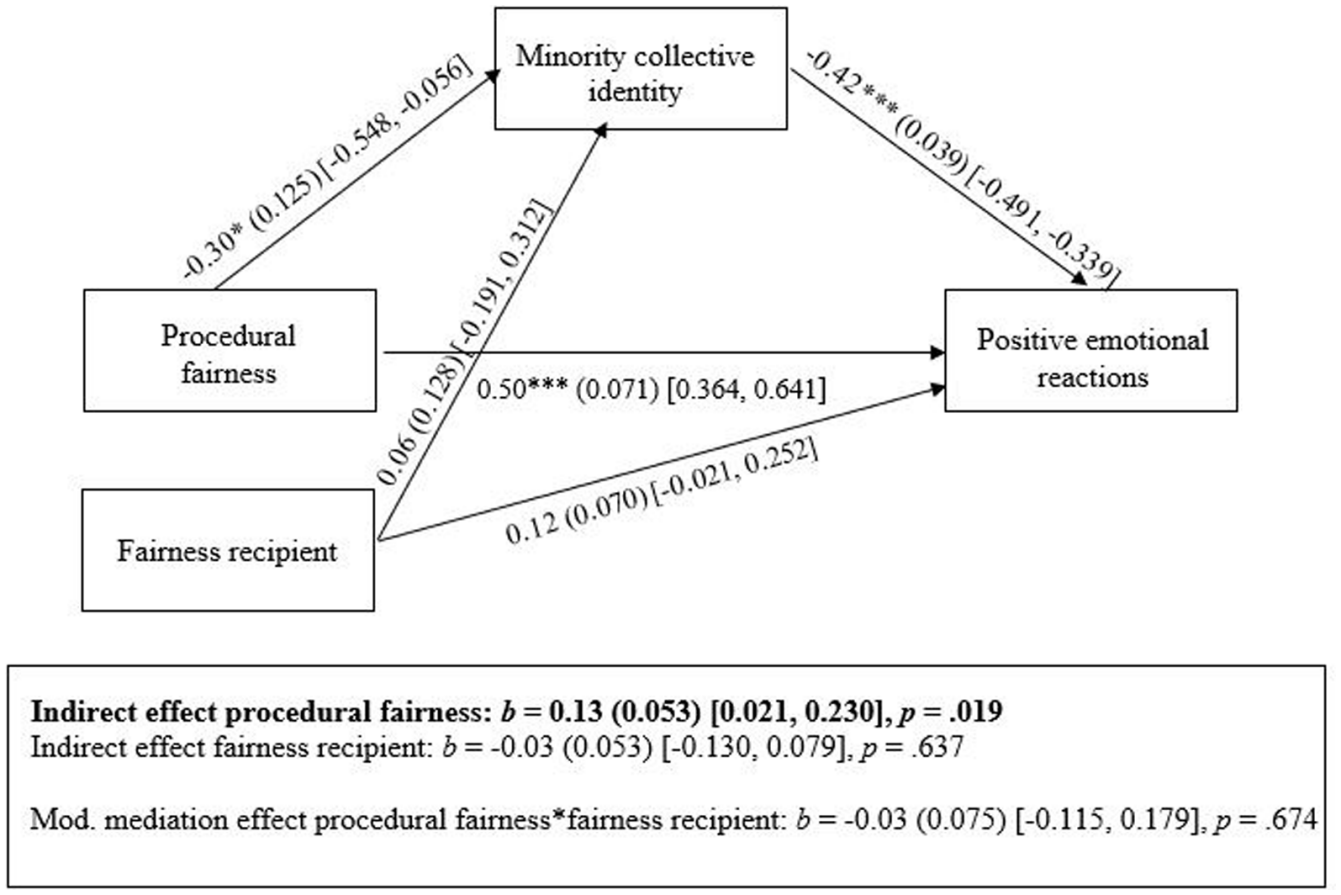
Results of moderated mediation analyses Study 1. Procedural fairness (1  =  fair procedure, 0  =  unfair procedure) and fairness recipient (1  =  own minority group, 0  =  other minority group) were dummy-coded. Insignificant moderation and moderated mediation pathways are not shown, for sake of simplicity. Mod. mediation effect  =  moderated mediation effect of procedural fairness*fairness recipient (via minority collective identity) on positive emotional reactions. *p  <  0.05, ***p  <  0.001.

Lastly, to investigate if procedural fairness effects are somewhat smaller when another minority group is the fairness recipient (compared to the own minority group), we added a term encoding the hypothesized procedural fairness*fairness recipient interaction to the model. The results revealed, however, that this interaction did not reach significance [*b* = −0.08 (−0.430, 0.278), *SE* = 0.180, *p* = 0.673], nor did the moderated mediation pathway from the interaction term (through minority collective self) to positive emotional reactions [*b* = 0.03 (−0.115, 0.179), *SE* = 0.075, *p* = 0.674]. See Figure 2 for an overview of the final moderated mediation model and all the relevant pathways.

### 3.3. Discussion

The results of Study 1 were mainly in line with our hypotheses. Firstly, and corroborating prior research (Dierckx et al., 2020, 2021; Valcke et al., 2020a,b), it was shown that the procedurally fair resolution of an ethnic-cultural issue by a societal actor elicited more positive reactions among minority group members, compared to a situation where this issue was dealt with in a procedurally unfair way. As such, these results are further indicative of the potential of procedural fairness in ethnic-cultural decision-making as a tool to foster social cohesion. Secondly, and more importantly, it was observed that minority collective identity mediated the relationship between procedural (un)fairness and participants’ positive emotional reactions. Interestingly, both our frequentist and our Bayesian analyses yielded no evidence for differential effects of our fairness manipulation across fairness recipient groups. In other words, our results did not deliver any evidence that minority group members respond differently when the fairness beneficiary is a member of their own vs. another minority group. These results thus align with our “generalized” version of the collective model, which states that procedurally fair treatment of minority groups impacts upon minority group members because of the shared attribute of being a minority member as such.

Study 2 set out to satisfy three research questions. First, we aimed to gauge majority group member reactions to ethnic-cultural fairness and compare them with those of members of the involved minority group. To this end, we sampled both majority (White Americans) and minority group members (Black Americans), and investigated their reactions to a paradigm similar to Study 1 (i.e., fair vs. unfair treatment of a Black American by a societal actor). Secondly, we investigated the role of universal and specific moral obligations as explanatory variables for majority group members’ observed responses. Moreover, given that half of our sample consisted of minority members, we also administered our Study 1 measure of (minority) collective identity. As such, Study 2 served as an additional test of the collective model.

## 4. Study 2

### 4.1. Method

#### 4.1.1. Participants

Study 2 was preregistered at https://aspredicted.org/QVT_1MG. Sample size was determined prior to data collection. Our main aim was to study majority group members’ responses to procedural fairness vis-à-vis an ethnic-cultural issue, and to unravel the psychological mechanisms underlying these reactions. However, we were not aware of any comparable studies in literature from which an effect size guideline could be derived. As such, given that Study 2 additionally served as a replication of the effects of (minority) collective self as an explanatory variable obtained in Study 1, we based our sample size calculations on this study objective. Hence, we simulated 10,000 times a model wherein (minority) collective self mediated the relationship between procedural fairness and emotional reactions (in the minority group subsample). The Study 1 regression coefficients were used as estimates for the direct paths in the simulations. The results of these calculations revealed that, assuming standard criteria (*α* = 0.05), a sample of *N* = 300 participants would yield over 99% power to detect an indirect effect of the aforementioned magnitude.^4^ Anticipating some dropout, we aimed to oversample by 10% and thus intended to recruit 660 Prolific workers (330 White American majority members^5^ and 330 Black American minority group members).^6^ However, the size of each subsample was also contingent upon participant availability on Prolific, and study completion among Black minority group members proceeded at a slower pace than among their White majority counterparts.^7^ Therefore, at the (preregistered) end of the data collection period – i.e., 2 weeks after the launch – only 218 Black minority group members had completed the study (versus 331 White majority group members). A further 40 participants were excluded because they failed the complainant’s ethnic background attention check (“What was the ethnic group of the complainant?”). Hence, our final sample consisted of 509 participants (306 White majority group members, 181 males, *M* = 37.66, *SD* = 14.59, range = 18–91).

#### 4.1.2. Procedure

All Prolific workers were invited to participate in a study about “social opinions.” They then read a fictitious news article describing the court case of a Black U.S. citizen who was suing a White landlord for alleged discrimination. Specifically, the article conveyed that the landlord had refused the complainant as a tenant of one of his houses. To further arouse participants’ suspicions that discrimination was indeed involved, two elements were added to the story. First, participants read that the landlord had added the man on Facebook the day before the refusal. Secondly, it was clarified that the house still appeared to be for rent. Then, participants were informed about the decision procedures implemented by the judge to reach his verdict. Within each ethnic subsample (White vs. Black), participants were then randomly assigned to either the fair or the unfair condition. As such, four experimental conditions were created, based on participant ethnic background (White vs. Black) and procedural fairness manipulation (fair vs. unfair): White/fair (*n* = 154), White/unfair (*n* = 152), Black/fair (*n* = 102), and Black/unfair (*n* = 101). As before, in the fair procedure conditions, participants read that the judge had allowed both the complainant and the defendant to voice their views and to present the reasons behind them. Conversely, in the unfair procedure conditions, participants were informed that the judge had refrained from making such efforts. Following the manipulation, participants completed three manipulation checks, our attention check and dependent measures. Finally, they were debriefed and thanked for their cooperation.

#### 4.1.3. Measures

To gauge participants’ *positive emotional reactions* to the news article, we implemented the same items as before (*M* = 2.02, *SD* = 0.81, *α* = 0.83). Participants were further asked to indicate for a series of statements the extent to which they applied as a reason for their (lack of) positive emotional reactions (scaled 1 = “not applicable at all” to 5 = “strongly applicable”). Amongst some filler items, we included three items referring to the *specific moral obligation to act rightfully toward disadvantaged groups*: “The news article made me feel this way because… [the complainant] belongs to a group with limited social power and status, and care should be exercised when dealing with people like him,” “… [the complainant] belongs to a group with limited social power and status, and representatives of the U.S. institutions have the obligation to behave morally and rightfully toward people like him,” and “… [the complainant] belongs to a group with limited social power and status, and representatives of the U.S. institutions must take care to behave tolerantly toward people of such disadvantaged groups” (*M* = 3.48, *SD* = 1.20, *α* = 0.85). Three further items referring to the *universal moral obligation to act rightfully*: “[the complainant] is a human being, regardless of which ethnic group he belongs to, and is therefore deserving of fair and moral treatment,” “All humans should be treated respectfully, and the court should thus treat [the complainant] fairly and morally, regardless of whether he is a minority member or not,” and “I care a lot about how all people are treated, and the story of [the complainant] reminds me that American courts should do better to serve humanity, regardless of status or ethnic differences” (*M* = 4.70, *SD* = 0.64, *α* = 0.84). Furthermore, in the Black subsample, we also administered the six items referring to participants’ *collective self* as a reason to feel positive emotional reactions (*M* = 3.88, *SD* = 0.86, *α* = 0.80).

### 4.2. Data analysis and results

#### 4.2.1. Manipulation checks

A two-way ANOVA with procedural fairness (fair vs. unfair) and fairness recipient (own vs. other minority group) on the first manipulation check (“According to the news article, the decision was made in a fair and unbiased way”) revealed a main effect of procedural fairness [*F* (1, 504) = 697.44, *p < 0*.001, *η^2^* = 0.58]: Participants in the fair procedure conditions (*M* = 4.52, *SD* = 0.97) displayed stronger agreement with this statement compared to those in the unfair procedure conditions (*M =* 1.78, *SD* = 1.36, *d* = 2.33). Unexpectedly, a main effect of participant ethnic background was also found [*F* (1, 504) = 4.70, *p = 0*.031, *η^2^* = 0.01]: White participants (*M* = 3.07, *SD* = 1.82) displayed less agreement with this statement than Black participants (*M =* 3.30, *SD* = 1.79, *d* = 0.12). The procedural fairness*participant ethnic background interaction did not reach significance (*F* = 3.74, *p* = 0.054).

Furthermore, for the second item (“According to the news article, the judge allowed the complainant to voice his opinions”) participants were also more likely to agree in the fair procedure conditions (*M =* 4.65, *SD* = 0.92) vs. the unfair procedure conditions [*M =* 1.50, *SD* = 0.92, *d =* 3.43; *F*(1, 504) = 1491.31, *p < 0*.001, *η^2^ = 0*.75]. Neither the main effect of participant ethnic background, nor the interaction reached significance (both *F*s < 2.89, *p*s > 0.089).

Lastly, for the third item (“The news article referred to the decision by a District Court not to prosecute a landlord accused of discrimination”), we obtained an unexpected main effect of participant ethnic background [*F* (1, 504) = 4.17, *p* = 0.042, *η^2^* = 0.01]: White participants (*M* = 4.80, *SD* = 0.60) displayed stronger agreement with this statement compared to Black participants (*M =* 4.68, *SD* = 0.72, *d* = 0.19). Neither the main effect of procedural fairness, nor the procedural fairness*participant ethnic background interaction reached significance (both *F*s < 0.65, *p*s > 0.422). As such, we concluded that our manipulation had been successful.

#### 4.2.2. Dependent variables

We first explored participants’ emotional reactions after reading the news article and their references to specific moral obligations to act rightfully (toward disadvantaged groups) and universal moral obligations to act rightfully as reasons to have positive emotional reactions. Overall, participants reported rather negative emotional reactions [*M* = 2.02, *SD* = 0.82, *t* (506) = −13.37, *p* < 0.001]. Similarly, participants’ ratings of specific [*M* = 3.48, *SD* = 1.20, *t* (505) = 18.39, *p* < 0.001] and universal moral obligations to act rightfully [*M* = 4.70, *SD* = 0.64, *t* (505) = 78.88, *p* < 0.001] were significantly above average. Furthermore, for the Black subsample, the ratings of collective self as a reason for emotional reactions were also significantly above-average [*M* = 3.88, *SD* = 0.86, *t* (202) = 22.78, *p* < 0.001].

To further investigate the effects of our manipulations, we ran a two-way MANOVA with procedural fairness (fair vs. unfair) and participant ethnic background (White vs. Black) as between-subject factors, and specific moral obligations, universal moral obligations, and positive emotional reactions as the dependent variables. The omnibus MANOVA test yielded a significant main effect of procedural fairness [*F* (1, 500) = 27.69, *p* < 0.001, *η^2^* = 0.14]. Follow-up Welch’s *t*-tests revealed significant differences between the fair procedure and the unfair procedure conditions for specific moral obligations [*M*_fair_ = 3.33, *SD*_fair_ = 1.21; *M*_unfair_ = 3.63, *SD*_unfair_ = 1.17; *F* (1, 504) = 7.94, *p* < 0.001] and positive emotional reactions [*M*_fair_ = 2.31, *SD*_fair_ = 0.84; *M*_unfair_ = 1.72, *SD*_unfair_ = 0.67; *F*(1, 484) = 75.90, *p* < 0.001], but not for universal moral obligations (*p* = 0.529). Likewise, the omnibus MANOVA test yielded a significant main effect of participant ethnic background [*F* (1, 500) = 8.63, *p* < 0.001, *η^2^* = 0.05]. Follow-up *t*-tests revealed significant differences between the White and the Black subsample for specific moral obligations [*M*_White_ = 3.29, *SD*_White_ = 1.26; *M*_Black_ = 3.76, *SD*_Black_ = 1.02; *F* (1, 487) = 21.00, *p* < 0.001] and positive emotional reactions [*M*_White_ = 2.11, *SD*_White_ = 0.84; *M*_Black_ = 1.87, *SD*_Black_ = 0.75; *F* (1, 465) = 38.60, *p* < 0.001], but not for universal moral obligations (*p* = 0.983). The interaction term between procedural fairness and participant ethnic background did not reach significance, *F* = 0.37, *p* = 0.774.

For the Black subsample, we ran an additional ANOVA with procedural fairness as between-subjects factor and collective self as the dependent variable. The results of this analysis revealed a significant effect of procedural fairness, *F* (1, 201) = 5.85, *p* = 0.016, *η^2^* = 0.03. Participants in the fair procedure condition (*M* = 3.74, *SD* = 0.94) were less likely to rate collective self as a reason for positive emotional reactions than those in the unfair procedure condition (*M =* 4.03, *SD* = 0.75, *d* = 0.34).

#### 4.2.3. Bayesian analysis

To further substantiate our claim that ethnic-cultural procedural fairness should sort similar effects among majority group members, compared to their minority counterparts (i.e., *Hypothesis 3*), we additionally calculated *BF*s for the critical procedural fairness*participant ethnic background interactions (dependent variables = specific moral obligations, universal moral obligations, positive emotional reactions). The results of these analyses revealed that the *BF*s associated with these interaction effects were 0.14, 0.19, and 0.19, respectively. Or, in other words, the *BF*s favoring the null hypothesis were 7.14, 5.26 and 5.26, suggesting substantial evidence for the null hypothesis. Thus, taken together with our frequentist results, our Bayesian analyses further validated our expectation that ethnic-cultural procedural fairness should sort similar effects among majority group members (compared to their minority counterparts).

#### 4.2.4. Mediation analysis

To investigate the role of specific and universal moral obligations as explanatory process variables, we conducted a mediation analysis, using the *Lavaan* package in R, with procedural fairness (dummy-coded: fair = 1, unfair = 0) and participant ethnic background (White = 1, Black = 0) as independent variables, specific and universal moral obligations as mediators and positive emotional reactions as outcome. In line with our hypothesis, the results of this analysis indeed revealed that specific moral obligations mediated the relationship between procedural fairness and positive emotional reactions [*b* = 0.07 (0.018, 0.113), *SE* = 0.024, *p* = 0.006]. The indirect effect of procedural fairness on positive emotional reactions through universal moral obligations did not reach significance [*b* = 0.01 (−0.024, 0.047), *SE* = 0.018, *p* = 0.529]^8^.

#### 4.2.5. Moderated mediation analysis

To investigate the hypothesis that procedural fairness effects are somewhat smaller among majority group members (compared to minority group members), we added terms encoding the hypothesized procedural fairness*participant ethnic background interactions to the model and regressed them on our hypothesized mediators (i.e., specific and universal moral obligations). The results revealed, however, no significant interactions (both *b*s < |0.10|, both *p*s > 0.420), nor did any of the moderated mediation pathways from the interaction terms (through the mediators) to positive emotional reactions reach significance (both *b*s < |0.04|, both *p*s > 0.423).

#### 4.2.6. Exploratory mediation analysis on black subsample

**Figure 3:**
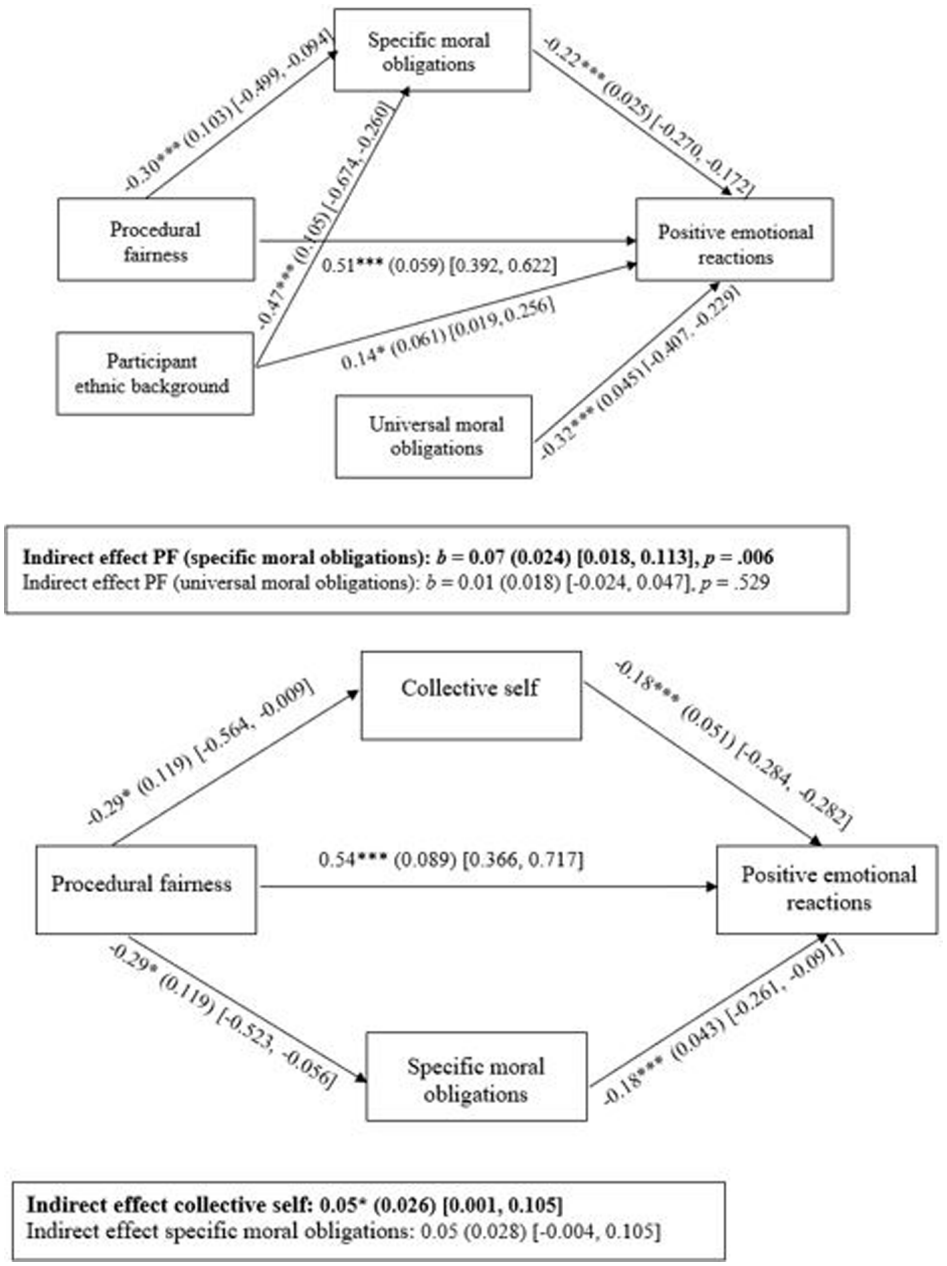
Results of mediation analyses on full sample (top panel) and on Black subsample (bottom panel), Study 2. Procedural fairness (1  =  fair procedure, 0  =  unfair procedure) and participant ethnic background (1  =  White, 0  =  Black) were dummy-coded. Only significant pathways are shown. PF = procedural fairness. *p  <  0.05, ***p  <  0.001.

Lastly, we also gauged the relative contribution of specific moral obligations and collective self in the effect of procedural fairness on positive emotional reactions in the Black subsample. To this end, we pitted both variables against each other in a final mediation analysis with procedural fairness as independent variable, specific moral obligations and collective self as mediators and positive emotional reactions as outcome variable. The results of this analysis indeed revealed that collective self mediated the relationship between procedural fairness and positive emotional reactions [*b* = 0.05 (0.001, 0.105), *SE* = 0.026, *p* = 0.045]. The indirect effect of procedural fairness on positive emotional reactions through specific moral obligations did not reach significance [*b* = 0.05 (−0.004, 0.105), *SE* = 0.028, *p* = 0.070]^9^. See Figure 3 for an overview of the (moderated) mediation models for the full and Black (sub)sample and all the relevant pathways.

### 4.3. Discussion

The results of Study 2 were mainly in line with our hypotheses. Firstly, both our frequentist and our Bayesian analyses revealed no evidence for dissimilar reactions to procedurally fair treatment of a Black American minority group member among White participants, compared to Black participants. Furthermore, our mediation analyses showed that participants’ reactions were primarily driven by perceived moral obligations to act rightfully and unbiasedly toward disadvantaged groups. As such, our results seem to favor an explanation of majority member reactions in terms of specific moral obligations, rather than universal moral obligations (to act rightfully and unbiasedly toward all humanity) – which was shown to be only a weak and secondary driver of the observed effects. Conversely, for the Black subsample, it was found that collective identity was the principal explanatory variable, when entered simultaneously with both types of moral obligations into our mediation model.

## 5. General discussion

The present research investigated the reactions of members of uninvolved minority groups and majority groups, when they witness societal actors dealing in a procedurally (un)fair way with ethnic-cultural issues (i.e., issues related to ethnic-cultural, religious or linguistic matters). Drawing on Valcke et al.’s (2020b) collective model of fairness, we hypothesized that uninvolved minority group members would display similar responses (compared to members of the involved minority group), and that these would be driven by a shared bond with the fairness recipient(s) based on minority group membership as such (i.e., minority collective identity). Furthermore, we also theorized that majority group members would display similar responses (compared to members of the involved minority group), and that these reactions would be grounded in (universal and/or specific) moral obligations. The results of two experiments were in line with our predictions. In Study 1, no evidence was obtained for dissimilar emotional reactions among Hispanic Americans when a fellow Hispanic American was treated (un)fairly, compared to when a Black American was treated (un)fairly. It was further shown that minority collective identity mediated this relationship. In Study 2, no evidence was obtained for dissimilar emotional reactions among White and Black Americans when a Black American was treated (un)fairly. Our analyses further revealed that, whereas these reactions were shaped by the perceived moral obligation to act rightfully toward disadvantaged groups among White majority participants, Black minority participants’ reactions were chiefly driven by Black collective identity – and, to a lesser extent, by specific moral obligations.

### 5.1. Theoretical contributions

The present research makes various contributions to the scholarly literature on ethnic-cultural procedural fairness. First and foremost, our results both provide corroboration and a noteworthy extension of the collective model (Valcke et al., 2020a,b). That is, in Study 2, Black American participants displayed above-average agreement with references to being a Black minority group member themselves, as a reason for their emotional involvement with the procedurally (un)fair treatment of a fellow Black American citizen. More importantly, their Black American minority identity was also shown to be a key explanatory variable for their emotional reactions. As such, the present research thus contributes to the recent literature on ethnic-cultural procedural fairness (Dierckx et al., 2020, 2021, 2023a,b; Valcke et al., 2020a,b) by providing an additional empirical test of the underlying assumptions of the collective model. Moreover, our results also characterize and clarify the reactions of members of *uninvolved* minority groups. Specifically, the Study 1 findings show that positive emotional reactions to ethnic-cultural procedural fairness “generalize” to members of minority groups that are not directly involved in the ethnic-cultural issue under scope. And, critically, these analogue reactions can be explained in terms of a “common” minority identity, that is, as a consequence of a liaison between the fairness recipient and witness based on minority group membership as such. A parallel with social categorization literature (Brewer, 1979; Tajfel and Turner, 1979; Brown and Turner, 1981; Turner et al., 1987), and in particular the common ingroup identity model (Gaertner et al., 1993) therefore imposes itself. This influential theoretical framework asserts that intergroup bias can be successfully reduced when one transforms his/her representation of two groups (i.e., the ingroup and a given outgroup) into a single, “superordinate” group (Gaertner and Dovidio, 2000, 2005). Mirroring these processes, our results revealed that positive emotional reactions to procedurally fair treatment of members of other minority groups were driven by the salience of a single, superordinate “minority identity,” which encompasses both ingroup (e.g., fellow Hispanic Americans) and outgroup members (e.g., Black Americans) in a single social group representation (i.e., “members of a minoritized group”). As such, it appears that increasing the salience of existing common superordinate group memberships is a promising way to foster social cohesion, not only because it can reduce intergroup bias (as in Gaertner et al., 1993), but also because it increases intergroup solidarity and empathy (as in Study 1).

Furthermore, another comparison which imposes itself is between the collective model and the group-value model of procedural fairness by Lind and Tyler (1988). This theoretical framework holds that fair treatment by authorities communicates the symbolic message to its recipients that their group is valued (i.e., “pride,” Tyler et al., 1996) and that they themselves are respected and accepted (i.e., “respect,” Lind and Tyler, 1988; Tyler and Lind, 1992). These assumptions align closely with two of the central tenets of the collective model. Most relevant to the present contribution, follow-up empirical work on the group-value model by Smith and Tyler (1997) has revealed that these procedural fairness effects emerge both for smaller more salient groups (e.g., gender) and for broader social categories (e.g., shared interests). The present findings corroborate these scholars’ observations, revealing positive emotional reactions to treatment fairness targeting both a smaller and more differentiated group (i.e., one’s own minority group) and a broader social category (i.e., another minority, and thus, minority groups in general). In addition, our results reveal *why* these effects occur, that is, as a natural consequence of the fact that minority group membership of broader social categories is inherently embedded into the collective self – which is made salient because ethnic-cultural issues, by definition, concern minority groups.

Moreover, the present research also contributes to literature by empirically scrutinizing the reactions of majority group members to ethnic-cultural procedural fairness. Specifically, Study 2 did not yield any evidence that White participants’ emotional reactions to the procedurally (un)fair treatment of a Black person by a societal actor are different from those of Black participants. At first sight, these findings align with the deontic model of justice (Folger, 1998, 2001), which posits that fairness is considered “an end in itself” (Beugré, 2012), and that people will positively appraise all behavior conforming to universal moral standards (to act rightfully and unbiasedly; Rupp and Bell, 2010). However, by gauging participants’ views on moral obligations, we were able to further unravel the specific psychological processes that gives rise to majority group member reactions to ethnic-cultural procedural fairness. That is, our results revealed that majority member responses were in fact mainly driven by a perceived moral obligation to behave ethically and rightfully toward members of “weaker,” disadvantaged groups, rather than by universal, deontic norms (i.e., to behave rightfully “towards humanity,” and thus, toward members of *all* societal groups). As such, the present results provide a more fine-grained, nuanced picture of majority third parties’ responses to ethnic-cultural procedural fairness; and, in doing so, they further refine Folger’s (1998, 2001) deontic account of justice. Moreover, they align with recent research underscoring the importance that majority group members attach to “protecting the weak’,” to guide and evaluate their own behavior and that of fellow group members when they interact with lower-status groups (Dierckx et al., 2022). Relatedly, the present examination further unraveled that such specific moral obligations also play a role in the reactions of minority group members to ethnic-cultural procedural fairness – an explanation which was not anticipated by the collective model, but nonetheless seems intuitive. Yet, by pitting collective identity and specific moral obligations against each other in the Study 2 mediation analyses, we were able to demonstrate that collective identity in particular is the key explanatory mechanism by which ethnic-cultural procedural fairness sorts its beneficial effects. As such, the present contribution additionally attests to the robustness of Valcke et al.’s (2020a,b) collective model.

### 5.2. Practical implications for social cohesion

The present results also have some important practical consequences. First and foremost, they implicate that the benign effects of ethnic-cultural procedural fairness could extend beyond what has been assumed so far by the collective model. As we outlined in the Introduction, the perception of fair resolution of ethnic-cultural issues by societal actors has been associated with a wide array of outcomes that are beneficial to social cohesion, e.g., enhanced sense of societal belonging and societal identification; Valcke et al. (2020a,b), community prosperity (e.g., increased social trust and trust in the national majority group; Dierckx et al., 2021), and personal well-being (e.g., mental health and life satisfaction, Valcke et al., 2020b), among members of the fairness recipient minority group. In this regard, it is thought-provoking that members of uninvolved minority groups displayed similar positive emotional reactions. And, we also note that our results revealed analogue psychological processes (i.e., minority collective identity) underlying these reactions. Taken together, these observations seem to suggest that uninvolved minority group members’ responses to ethnic-cultural fairness may not remain limited to sheer “passive” empathy but, by contrast, could entail consequences for social cohesion that are similar to the ones described above (e.g., societal identification). An explicit test of the latter premise fell out of the scope of the current study however, and remains yet to be tested. Nonetheless, to the very least, it appears that, by embedding procedural fairness into the decision procedures regarding ethnic-cultural issues, societal actors might be able to “kill two birds with one stone” and foster societal bonding among both fairness recipient and non-recipient minority groups.

A second practical implication relates to the positive emotional reactions observed among majority members in Study 2 – which were comparable to those of their minority counterparts. These benign responses can be seen as somewhat surprising and in contrast with prior empirical findings, which have consistently revealed that majority members tend to oppose diversity policies because they perceive them as harming their own interests (e.g., Plaut et al., 2011; Craig and Richeson, 2014; Brown and Jacoby-Senghor, 2021). Based on these findings, one would expect the fair resolution of ethnic-cultural issues to be a “zero-sum” game for social cohesion, whereby the positive effects (i.e., enhanced societal belonging and identification among the fairness recipient minority group) are nullified by the negative consequences (i.e., misapprehension among majority group members, deteriorated relations between minority and majority groups). The present results, however, suggest a more nuanced account. That is, majority group members may not necessarily oppose interventions designed to meet the needs of minority groups – provided that these decisions are made in an accurate, unbiased way. In this regard, we refer to the scholarly literature examining how affirmative action can be implemented without eliciting opposition by members of non-target groups (e.g., Nacoste, 1994), which has led to similar conclusions. At any rate, the present results thus inform societal actors that they should not refrain from ethnic-cultural procedural fairness because of concerns about majority member spite, and that the implementation of procedural fairness in the resolution of ethnic-cultural issues may yield “net” positive effects for social cohesion (i.e., positive responses among minority group members and neutral-to-positive responses among majority group members).

### 5.3. Limitations and directions for future research

A few limitations associated with the present studies should be considered. First, it should be noted that, for practical reasons (i.e., the 2020–2022 global Covid-19 pandemic), we limited our scope to online Prolific samples. Although crowdsourcing platforms are generally regarded a “useful method for conducting a wide range of research” (Buhrmester et al., 2018, p. 152) and a reliable data collection tool (Paolacci and Chandler, 2014), we acknowledge that sole reliance on survey panel-based samples to draw conclusions about the general population should be avoided. We therefore strongly encourage future research using various types of other data (e.g., convenience samples, student pools, peer reports) to study third-party reactions to ethnic-cultural procedural fairness enactment.

Secondly, we believe that further empirical attention for the reactions of other, non-involved minority group and majority group members to ethnic-cultural fairness enactment is warranted. In the presently reported studies, the issues under scope – e.g., procedurally fair treatment of a Black citizen in a court case – did not affect nor hurt the interests of other groups. However, it remains yet to be shown that similar positive emotional reactions would be observed for decisions wherein the interests of fairness recipient groups and non-involved minority and majority groups are directly opposed. See for example the ban imposed in 2019 by the Belgian government on the Muslim and Jewish ways of ritually slaughtering animals, whereby the religious interests of both these minority groups were in clear discordance with the demands of – predominantly Belgian majority – members of animal rights groups (Schreuer, 2019). Or consider the implementation of affirmative action initiatives such as university admission programs which increase the acceptance of underrepresented minority groups or priority housing projects for refugees –diversity efforts which have been shown to be perceived by majority group members as strongly at odds with their own interests (Plaut et al., 2011; Brown and Jacoby-Senghor, 2021). Future research aimed at uncovering potential “boundaries” on benign reactions of fairness witnesses could explicitly focus on procedural fairness perceptions vis-à-vis these and similarly sensitive issues. Needless to say, such research endeavors would undoubtedly help to characterize the robustness of the observed effects.

Thirdly, it should be acknowledged that both the Hispanic and the Black American community are highly heterogenous in nature. Specifically, while minority members belonging to these groups may come from Latin America or Africa, they may nonetheless also have other (e.g., Spanish, Italian, French, Kenyan, South African, etc.) backgrounds. And, it can reasonably be expected that such variations in ethnic-cultural background could further qualify third-party responses to ethnic-cultural procedural fairness. Future research could explicitly take such within-group variability into account, in order to provide a more fine-grained analysis of ethnic-cultural procedural fairness effects on members of uninvolved minority groups.

Lastly, we must stress that the introductory sentence in the questionnaires measuring our mediating variables explicitly referred to participants’ emotional reactions. We can therefore not rule out the possibility that this choice of wording may have inflated the magnitude of the relationship between the mediators and the dependent variable. As such, we encourage future researchers to implement other operationalizations of minority collective self, specific moral obligations, and universal moral obligations, in order to verify the robustness of the relationship between these variables and bystander positive emotional reactions to ethnic-cultural procedural fairness.

### 5.4. Concluding remarks

The present contribution investigated third-party reactions to procedurally fair treatment of minority groups by societal actors, which has been shown to be a useful strategy to strengthen and foster social cohesion. Specifically, we examined the reactions of members of uninvolved minority groups and the majority group when they witness an instance of ethnic-cultural fairness. The results of two experiments revealed positive emotional reactions among both groups, comparable to the responses of minorities that are directly targeted by the fair treatment. Our findings further elucidated that, whereas minority group member reactions are primarily shaped by a “common minority identity,” majority group members’ responses are, conversely, driven by perceived moral obligations to “protect the weak.” Taken together, the present contribution thus further demonstrates that procedural fairness can be implemented by societal actors to successfully promote social cohesion among members of all societal groups.

## Data availability statement

The datasets presented in this study can be found in online repositories. The names of the repository/repositories and accession number(s) can be found at: https://osf.io/jv9gh/.

## Ethics statement

The studies involving human participants were reviewed and approved by the Faculty Ethical Board (Faculty of Psychological and Educational Sciences, Ghent University). The patients/participants provided their written informed consent to participate in this study.

## Author contributions

KD and AH analyzed and interpreted the data. KD provided a first draft of the article. AH, KB, and BV made critical revisions of the article. All authors gave their final approval of the version to be published and were involved in the conception and design of the work and the empirical studies.

## Funding

This work was supported by the Special Research Fund of Ghent University (Grant No. BOF16/GOA/007).

## Conflict of interest

The authors declare that the research was conducted in the absence of any commercial or financial relationships that could be construed as a potential conflict of interest.

## Publisher’s note

All claims expressed in this article are solely those of the authors and do not necessarily represent those of their affiliated organizations, or those of the publisher, the editors and the reviewers. Any product that may be evaluated in this article, or claim that may be made by its manufacturer, is not guaranteed or endorsed by the publisher.

## Footnotes

^1^ https://osf.io/jv9gh/

^2^Participants were included in the sample if they responded (1) “United States” to the prescreening question “What is your nationality” and (2) if they responded “Hispanic” to the prescreening question “Please indicate your ethnicity (i.e., peoples’ ethnicity describes their feeling of belonging and attachment to a distinct group of a larger population that shares their ancestry, colour, language or religion)?.”

^3^For exploratory purposes, we investigated an alternative explanation for the relationship between ethnic-cultural procedural fairness perceptions and positive emotional reactions. Specifically, it could be argued that the observed effects were *dependent on*, rather than *driven by* minority collective identity. For example, it seems conceivable that when being a minority group member is a central aspect of one’s collective identity, one would be more likely to respond positively when another minority group is treated fairly, compared to a situation wherein being a minority group member is only a peripheral aspect of one’s collective identity. As such, to explore the possibility that minority collective identity is a *moderator* rather than a *mediator* variable of ethnic-cultural procedural fairness effects, we ran a one-way ANOVA with procedural fairness (fair vs. unfair) and fairness recipient (own vs. other minority group) as between-subject factors, minority collective self as a moderator, and positive emotional reactions as the dependent variable. Critically, we added an additional procedural fairness*minority collective identity interaction term to this model. This interaction, however, did not reach significance (*p* = 0.565).

^4^Results of an additional sensitivity analysis further revealed that, given this sample size and the observed relationships between our focal variables in the majority group subsample, our study had 80% power to detect minimal slopes of size *b =* 0.09–0.18 for any of the hypothesized mediators underlying the procedural fairness effect in the majority group.

^5^Participants were included in the sample if they responded (1) “United States” to the prescreening question “What is your nationality” and (2) if they responded “White/Caucasian” to the prescreening question “Please indicate your ethnicity (i.e., peoples’ ethnicity describes their feeling of belonging and attachment to a distinct group of a larger population that shares their ancestry, colour, language or religion)?.”

^6^Participants were included in the sample if they responded (1) “United States” to the prescreening question “What is your nationality” and (2) if they responded “Black/African American” to the prescreening question “Please indicate your ethnicity (i.e., peoples’ ethnicity describes their feeling of belonging and attachment to a distinct group of a larger population that shares their ancestry, colour, language or religion)?.”

^7^Note that the availability of White American Prolific workers (*N* = 25,431 who indicate being fluent in English in the prescreening survey) exceeds by far that of Black American workers (*N* = 2,793 who comply with the same criterion). The maximum sample size of the Black American subsample was further limited by the fact that we had already recruited *N* = 530 workers in Studies 2 and 3.

^8^Analogous to Study 1, we investigated the possibility that (specific and universal) moral obligations are *moderator* rather than *mediator* variables of ethnic-cultural procedural fairness effects. As such, we ran two one-way ANOVAs with procedural fairness (fair vs. unfair) and participant ethnic background (White vs. Black) as between-subject factors, moral obligations (ANOVA model 1: specific; model 2: universal) as moderators, and positive emotional reactions as the dependent variable. Critically, we added additional procedural fairness*moral obligations interaction terms to the models. These interactions, however, did not reach significance (both *p*s  >  0.170).

^9^Analogous to Study 1, we investigated the possibility that minority collective identity is a *moderator* rather than a *mediator* variable of ethnic-cultural procedural fairness effects. As such, we ran a one-way ANOVA with procedural fairness (fair vs. unfair) as between-subject factor, minority collective identity as moderator, and positive emotional reactions as the dependent variable. Critically, we added an additional procedural fairness*minority collective identity interaction term to the model. This interaction, however, did not reach significance (*p*  =  0.261).
